# A quantitative study of race and gender representation within London medical school leadership

**DOI:** 10.5116/ijme.609d.4db0

**Published:** 2021-05-27

**Authors:** Sophie Hoque, Elizabeth H. Baker, Adrienne Milner

**Affiliations:** 1 Barts and The London School of Medicine and Dentistry, Queen Mary University of London, UK; 2 Department of Sociology, the University of Alabama at Birmingham, USA; 3 Department of Health Sciences, College of Health, Medicine and Life Sciences, Brunel University London, UK

**Keywords:** Race, women, leadership representation, medical school

## Abstract

**Objectives:**

To explore potential disparities in representation of Racially Minoritised (RM) persons and women in leadership roles in London Medical Schools compared to their RM and female student populations.

**Methods:**

General Medical Council's Medical School Annual Return 2017-18 data and official leadership team webpages were used to determine percentages of RM and female students and percentages of RM and women leaders in London medical schools. Student and leadership team percentages were then compared using chi-squared tests to assess statistically significant differences.

**Results:**

The percentage of RM persons filling leadership roles in London medical schools combined was statistically significantly less than the percentage of RM persons that compose the combined student body (8.6% (N=81) versus 60.2% (N=8786, χ^2^(1, N=8,867)=88.83, p<0.001). There was no statistically significant difference between the percentage of women filling leadership roles and the percentage of women in the combined student body (43.4% (N = 83) versus 52.5% (N=9026, χ^2^(1, N=9,109) =2.85, p=0.0913).

**Conclusions:**

Results mirror the underrepresentation of RM persons in leadership positions throughout the National Health Service (NHS) and in higher education but reflect the improved representation of women in leadership positions seen at the NHS board level. Greater effort is necessary to rectify RM representation within London medical school leadership teams. This is especially imperative given that racially similar role models for RM students are an important predictor in determining academic and future success.

## Introduction

The COVID-19 pandemic has brought scholarly and public attention to racial health disparities in the United Kingdom with research demonstrating substantial negative health outcomes for Racially Minoritised (RM) persons compared to their white counterparts.^1^^,^^2^ Inequality in COVID-19 and other health outcomes, in part, may potentially be attributed to the lack of diversity within the National Health System (NHS), and especially in terms of a lack of representation of RM persons at the highest levels of the medical profession and in decision-making roles.^3^^,^^4^ Indeed, previous research has shown that diversity in the medical profession is a contributing component to the health of RM persons and women.^5^^,^^6^

Because medical schools are the first site for shaping future doctors, it could be argued that the decision-makers within these environments also are an important factor in public health outcomes. Thus, representation of RM persons and women in medical education leadership roles is vital to the success of RM and female students as they progress to clinicians. This paper seeks to explore the representation of RM persons and women in leadership roles in London Medical Schools by comparing the percentage of RM persons and women in leadership roles in London medical schools to that of their student populations. UK medical schools exist at the intersection of the NHS and higher education, training the doctors of the future by offering a vocational degree. London is a fitting location for the current analysis for three reasons: 1) it is one of the most racially diverse cities in the world; 2) there remains heightened racial diversity of medical students in London as compared to other medical schools in the UK; and, 3) London was ranked in 2018 and 2019 by Quacquarelli Symonds (QS) as the world's best student city.^7^^, ^^8^

There are five medical schools in London: Barts and The London School of Medicine and Dentistry (Barts), Kings College London GKT School of Medical Education (GKT), Imperial College School of Medicine (ICSM), St George's, University of London (St George's) and University College London Medical School (UCL). These institutions all express their commitment to equality and diversity as members of the Athena Swan Charter, with only St George's not a member of the race equality charter.^9^^-^^13^

Since the 1970s, the diversity of the UK medical student population with regards to race and gender has increased.^14^ Currently, medicine has one of the highest percentages of RM students compared to other undergraduate degrees.^15^ According to General Medical Council (GMC) data, 39% of students enrolled in medicine are of Mixed, Asian, Black or other Ethnic descent, while female students make up over half of the national cohort (N = 40997).^16^ The percentage of RM medical students is higher in London,^17^ reflecting the higher concentration of RM persons in the general population in the capital.^17^

While progress has been made in increasing RM and female admissions to medicine, the underrepresentation of women and RM persons in senior medical positions and leadership positions remain. It is a national policy that NHS leadership teams should reflect the diversity of the populations they serve. This is likely to improve the planning and provision of services and address disparities in deprived communities, including RM populations, which have traditionally been failed by the system.^18^ However, Jan Sobieraj, previous Managing Director of the NHS, argues that senior members of NHS organisations do not represent the populations they serve, and also they do not represent the staff that they manage.^18^ An investigation by the Labour party in 2016 highlighted how white males still dominate leadership positions in NHS England. Examining 1450 board members in 114 Trusts that responded to their Freedom of Information request, only 2% of Trust chairs were RM, and 28% were women. Although improvements by gender were noted at executive board level where women filled 47% of positions, only 4% of executive board roles were filled by RM persons. Furthermore, at a non-executive director level, only 7% of positions were filled by RM persons and 38% of positions were filled by women.^19^ These numbers reflecting leadership representation are in stark contrast to the highly racially diverse NHS workforce, with RM employees severely underrepresented at NHS board level.^20^

Underrepresentation of RM persons and women is also evident in senior positions across higher education. For example, AdvanceHE's report shows that only 9.6% of the 18,950 professors in the UK in 2018 were RM, and women made up less than a quarter of total professors (24.6%). When examining gender and race combined, 22.9% of these professors were white women, 2.1% were RM women, 7.5% were RM men, and 67.5% were white men.^21^ The Medical School Council's Survey of Medical Clinical Academic Staffing reflects similar results in clinical medical professorships, showing a decreased representation of women and RM persons with increased academic seniority.^22^

There are moral, legal, and public health justifications for ensuring representative leadership teams within the medical profession.^23^ Evidence shows that a diverse workforce, which values all staff member contributions, is associated with a higher quality of patient care.^3^ However, research suggests that currently, certain staff are valued more than others. For example, Kline's research^24^ highlights that NHS recruitment routinely favours white applicants, with white shortlisted candidates almost twice as likely to be appointed compared to shortlisted RM candidates. Because shortlisted candidates are likely to meet job selection criteria, this finding is not easily explained purely by candidate merit. Not only are RM candidates less likely to be selected for medical posts, Woolf and colleagues’ systematic review^25^ demonstrates that RM undergraduate medical students and doctors have poorer academic outcomes compared to their white counterparts throughout their careers. This systemic differential attainment^26^ is also reflected throughout higher education. There exists an awarding gap between the likelihood of white students and students from RM backgrounds achieving a first/upper second-class degree from UK universities,^24^ and this gap remains even after adjusting for multiple confounding factors including prior attainment, subject of study, age, and gender.^27^ In order to address the awarding gap, scholars have suggested that students require more racially similar role models in senior university positions.^28^^-^^31^

In terms of gender, studies suggest women outperform men during medical training^32^ and are more likely to receive an offer for speciality training.^33^ However, there is still a lack of women in medical leadership roles compared to their male counterparts. Although some scholars have attributed this to the 'glass ceiling' where there exist invisible barriers preventing women's promotion and career advancement,^34^^, ^^35^ studies have not systematically quantified gender disparities in representation.

To date, no studies have explored the race or gender make-up of medical school leadership teams in comparison to the student body. Furthermore, there is a lack of data specifically relating to the number of RM persons and women holding senior and leadership positions in medical schools. This study aims to address this gap in the literature by 1) quantifying the number of RM persons and women who hold leadership positions in five London medical schools and 2) compare the number of RM persons and women in these

roles to the numbers of RM and female students within their respective schools and across schools to determine potential disparities in race and gender representation.

## Methods

### Study design

To compare the percentage of RM persons and women in leadership roles in London medical schools to that of their student populations, both student data and leadership data was required. All data was collated in April 2019 from publicly available sources, and no identifying information has been used in the paper. Thus ethical approval was not required.

### Student data

The most recent, publicly available (2017/18) General Medical Council (GMC) Medical School Annual Return (MSAR) was used to quantify the percentage of RM students and female students in each of the five London medical school student populations. The GMC requires UK medical schools to complete the MSAR annually. The MSAR provides data on student numbers by various demographics, including gender and race. The data within the MSAR was self-reported by medical schools and was not checked against any other data source. The MSAR does not state how data was collected by each individual medical school, however, it is likely to be based on student self-declaration.^16^

To quantify the percentage of RM students at each medical school, the authors classified the following categories as RM to mirror the UK census: Mixed - White & Asian, Mixed - White & Black Caribbean, Mixed - White & Black African, Other Mixed, Black/Black British – African, Black/Black British – Caribbean, Black/Black British – Black Other, Asian/Asian British – Pakistani, Asian/Asian British – Indian, Asian/Asian British – Chinese, Asian/Asian British – Bangladeshi, Asian/Asian British - Other Asian, Other – Arab, Any Other. White British, White Irish, White Gypsy or Irish Traveller and White Other student numbers were classed as White. Where n<3 for a given category, data was excluded. Likewise, where race was unstated, these students were excluded from data analysis. 2.6% of medical students were excluded from the racial analysis because they did not report their race (N = 8786).

Percentages of RM students was then calculated. The percentage of female students at each London Medical School was readily available from the data as all students at each London medical school were classified as either 'female' or 'male.' This resulted in a total N=9026 for the gender analysis.

### Leadership team data

No existing data could be located that delineates the race or gender make up of medical school leadership teams in London medical schools. In order to quantify the race and gender of persons filling leadership positions in London medical schools, the authors used each medical school's official publicly available website to identify persons filling said roles in April 2019. The web pages listing each medical school's leadership team were identified from the results of a Google Search combining the name of each medical school and 'leadership', or in UCL's case, by navigating the school's website (N = 83).

The data collated includes 13 staff listed on Barts' leadership team webpage; 18 staff listed on ICSM's leadership team webpage; 23 staff listed on GKT's leadership team webpage; 25 staff listed on St George's leadership team webpage; and 4 staff listed on UCL's leadership webpage for whom photos could be located.

The first author, a medical student at the time, used the name and picture of medical leadership staff listed on each webpage to classify their perceived race (RM versus white versus unsure) and perceived gender (female versus male versus unsure). Using photos to identify demographic characteristics of target individuals is common in research investigating racial and gender inequality.^36^^-^^38^ Furthermore, it can be argued that beyond self-identification, the perceptions of potential and current medical students, other staff, and the general public are important in terms of representation.

We located photos using the following strategies: 1) Where pictures accompanied the name and position of individuals on the webpage, these were used to perceive the race and gender of said person. 2) Where names and positions existed as hyperlinks on the webpages, these were followed to the profiles of individuals. Where photos accompanied these profiles, these were used along with the given name to perceive the race and gender of said person. 3) Where only the name of individuals in leadership roles were listed (not as hyperlinks) on the webpages, or where hyperlinked profiles did not have a picture accompanying them, or where hyperlinks did not work, a Google search was carried out combining the individual's name as listed on the webpage and the medical school in which they were employed to locate a photo. Photos were located using the first page of 'all' Google results and/or the first 10 Google image results, with images published on the University website preferred.

Only individual images were used to discern the perceived race and gender of leadership staff to decrease error associated with identifying the wrong person in a picture. LinkedIn Profile Photos were considered acceptable where the position listed on the profile matched that on the university webpage. Black and white photos were accepted. All data where photos were not found (2 at GKT and 6 from St. George's) were excluded from the analysis.

Where the first author of the paper was unsure about the race and/or gender of an individual, an opinion from a second medical student was sought. There were two photos where race of the leadership team members was ambiguous (one from ICSM and one from St. George's). These were excluded from the RM analysis but included in the gender analysis. Sensitivity analyses were conducted to determine the extent that these exclusions substantively influenced our results. Because both student data and leadership team data was publicly available and no identifying information has been used in the paper, ethical approval was not required for this research.

### Comparison

The perceived percentage of RM persons filling leadership roles in each of the London medical schools was compared to the percentage of RM students in the corresponding student body. Likewise, the perceived percentage of women filling leadership roles in each of the London Medical schools was compared to the percentage of female students in the corresponding student body.

Statistical tests were carried out using MedCalc's online comparison of percentages calculator, which employs the Chi-Square method.^39^ However, the validity of using such a test on RM results for each individual medical school was questionable because the number of RM staff filling leadership positions was always less than 5. As such, RM data for all schools were also combined to give an overall result for London medical schools in total. Similarly, because of the low numbers of women leaders at Barts and UCL, gender data for all schools was also combined to give an overall result for London medical schools in total.

## Results

### Race

Results for the comparison of the percent of 'RM persons can be found in Table 1. The percentage of RM persons filling leadership roles in each individual London medical school is less than the percentage of students who are RM in each London medical school; differences in percentages ranged from 49% in GKT to 55.2% in UCL, where none of the leadership team was classed as RM. All results were found to be statistically significant at a level of 0.05. Combining data for individual medical schools strengthens the findings; the percentage of RM persons filling leadership roles in London medical schools combined is statistically significantly less than the percentage of RM persons that compose the combined student body (8.6% (N=81) versus 60.2% (N=8786), χ^2^(1, N =8,867) = 88.83, p<0.001), with the difference of 52% unlikely to be due to chance. The race could not be determined for ten individuals on the leadership team where pictures could not be located, or perceived race was ambiguous. Sensitivity analyses were conducted to determine if their inclusion in the RM category impacted our results. Chi-square tests indicate that even if those ten individuals were considered to all be RM, RM members are still drastically underrepresented in leadership positions given their percentage in the student population (χ^2^(1, N = 8,877) = 64.6, p<0.001).

**Table 1.** Comparison of the proportion of Racially Minoritised (RM) persons filling leadership roles in London medical schools to the proportion of RM students

School	Students		Leadership Team		% Difference in Proportion	95% Confidence Interval	Chi-square	p value
	Percent RM	N	Percent RM	N				
Barts and The London School of Medicine and Dentistry	58.2	1650	7.7	13	50.5	24.8 - 57.3	13.478	0.0002
Imperial College School of Medicine	64.3	1735	11.8	17	52.5	29.8 - 61.3	20.077	<0.0001
Kings College London GKT School of Medical Education	62	2292	13	23	49	29.8 - 57.7	23.084	<0.0001
St George's, University of London	61.2	1174	8.3	24	52.9	35.2 - 59.5	27.433	<0.0001
University College London Medical School	55.2	1935	0	4	55.2	6.2 - 57.4	4.914	0.0266
London Medical Schools Combined	60.2	8786	8.6	81	51.6	43.4 - 56.1	88.832	<0.0001

### Gender

Results for the comparison of percentages by gender can be found in Table 2. The percentage of women filling leadership roles at Barts is 23.1% (N = 13), while the percentage of students that are women is 52.8% (N=1686). This is the only gender result that was found to be statistically significant (χ^2^(1, N=1,699) = 4.56, p=0.0327). While the validity of the statistical test is questionable for Barts-specific data due to less than five women leaders in this school, it seems probable that the difference in percentages is not due to chance due to its size.

ICSM's and GKT's data showed that the percentage of women filling leadership roles is less than the percentage of female students. The differences in percentages are 13% (χ^2^(1, N = 1,860) =1.21, p = 0.271) and 17.3% (χ^2^(1, N = 2,357) =2.77, p=0.0961) respectively. However, results were not statistically significant. It is interesting to note that ICSM has one of the lower percentages of female students enrolled in medicine in the country, meaning less women would need to be part of their leadership team to represent the student population adequately.

The percentage of women filling leadership roles at St George's and UCL is higher than the percentage of female students; however, results are not statistically significant for either. For St George’s, the percentage of women holding leadership roles (60%) is similar to the percentage of female students (54.3%) in their respective populations (N=25 and N=1203, χ^2^(1, N=1,228)=0.32, p=0.5713). It should be highlighted that the size of the leadership team at UCL was only 4, meaning the 22.8 percentage point difference between the student and leadership female population is due to only one person on the leadership team (χ^2^(1, N=1,965)=0.83, p= 0.3619). Combining the data for individual medical schools shows that there is no significant difference in the percentage of women filling leadership roles in all London Medical Schools and the percentage of female students (43.4% (N= 83) versus 52.5% (N=9026), χ^2^(1, N=9,109)=2.85, p= 0.0913).

**Table 2:** Comparison of the proportion of women filling leadership roles in London medical schools to the proportion of female students

School	Students		Leadership Team		% Difference in Proportion	95% Confidence Interval	Chi-square	p value
	Percent Women	N	Percent Women	N				
Barts and The London School of Medicine and Dentistry	52.8	1686	23.1	13	29.7	2.4 - 44.8	4.561	0.0327
Imperial College School of Medicine	46.3	1842	33.3	18	13	-10 - 30.2	1.21	0.271
Kings College London GKT School of Medical Education	56.4	2334	39.1	23	17.3	-2.9 - 34.4	2.768	0.0961
St George's, University of London	54.3	1203	60	25	5.7	-13.8 - 22.5	0.321	0.5713
University College London Medical School	52.2	1961	75	4	22.8	-22.2 - 43.4	0.831	0.3619
London Medical Schools Combined	52.5	9026	43.4	83	9.3	-1.5 - 19.5	2.852	0.0913

## Discussion

Findings highlight that RM persons are significantly underrepresented in leadership positions within London medical schools when compared to the percentage of the student body that are RM. These results reflect trends found across NHS leadership^17^ and higher education.^21^ It is well established that persons tend to employ those who have similar characteristics to them, which may explain why these disparities remain^3^ as well as why the RM awarding gap in medical school persists.^28^^-^^31^

Findings suggest that women are not significantly underrepresented in leadership within London medical schools when compared to the percentage of the student body that are women. This suggests that progress has been made in ensuring women are represented in London medical school leadership, reflecting the trend now seen across NHS boards.^3^ It is possible that women, or perhaps more precisely white women, have progressed to becoming better represented in leadership teams within London medical schools while RM persons have not due to the timescales of commentary around issues of gender and racial diversity within the medical profession and higher education. While there is a large body of literature calling for better representation of women in medical leadership dating to the 1990s/2000s,^35^^,^^40^ it is only within the last ten years that combatting the racial awarding gap and racial discrimination within the NHS has become an essential part of the agenda.^3^

To our knowledge, this was the first study to quantify the number of RM persons and women who hold leadership positions in medical schools and compare the number of RM persons and women in these roles to the numbers of RM and female students to determine potential disparities in race and gender representation. It was not within the scope of this paper to examine the seniority or particular responsibilities of women within medical school leadership teams. Future research should examine whether leadership teams are meaningfully representative or if there has been a move to appoint more women to leadership positions for appearance sake. Furthermore, because this study relied on cross-sectional data, future research should examine whether gender equality remains a continued priority for London medical schools over time.  Additionally, the current study examines race and gender separately, even though these systems of oppression intersect with one another to affect outcomes.^41^ Future research should consider the intersection of race and gender in medical school leadership representation. It would be pertinent to compare the percentages of RM women filling leadership roles in medical schools to that of their student bodies. It is likely that RM women may be even more underrepresented than RM men, as seen in professorial roles in higher education.^21^ Publicly available data that combines the race and gender of the medical student population is not currently provided by the GMC's MSAR, and scholars should seek to ensure this data is collected and analysed to further understand potential differences in medical school leadership representation.

There are several methodological limitations of the study that should be noted. The creation of the leadership team dataset relied on the subjective perception of the first author as to whether an individual appeared RM versus white versus unsure. The perceived race of an individual may have conflicted with the self-identified race of an individual. However, it can be argued that perceived rather than self-identified race is more important in terms of a role modelling effect to current students, potential students, and other staff.

The authors were also limited in calculating the percentage of RM students by data collected and reported in the MSAR. The MSAR does not usually give exact figures for ethnic groups where n<3. Some students did not declare their ethnicity, giving rise to the 'unstated' category, so the percentages of RM versus white students may be slightly skewed. However, sensitivity analyses were conducted placing students whose race was unstated in either the RM or non-RM category and results remained virtually unchanged.

Moreover, the grouping of ethnic categories into RM versus white is a limitation because it ignores the race-ethnic heterogeneity of these two groups. Grouping data in this manner does not allow for the comparison of percentages of specific racial minorities. However, this was the most suitable method to allow for sensible statistical analysis considering the small number of persons comprising leadership teams and the even smaller number of RM persons in these teams. Moreover, on a practical level, it is easier to perceive someone's race as RM/not-white versus not-RM/white than to discern the intricacies of their racial background.

The authors were also limited in their analysis of gender by the data collected and reported in the MSAR, which assumed binary classification of gender: female versus male. It is unclear where transgender/non-binary students fit into the data or if medical schools provide an option for students to self-declare their gender as anything other than male/female. Classification of students into male/female informed the authors' decision to classify leadership team members in the same way.

## Conclusions

Although women have made progress in terms of their representation in leadership roles, RM persons are significantly underrepresented in London medical school leadership teams when compared to the corresponding percentage of RM students. The underrepresentation of RM persons in leadership roles has the potential for a significant impact on RM students, particularly when considering the link between the lack of racially similar role models and the RM awarding gap. Because persistent disparities in medical school hiring practices may thwart efforts to reduce the awarding gap between RM medical students and their white counterparts, greater effort is required to ensure leadership teams within London medical schools reflect the racial diversity of their students and do not disproportionally favour white candidates. London medical schools should seek to reduce racial bias in hiring and promotions through initiatives such as requiring those involved in decision-making processes to undertake anti-racism training and providing support for internal RM candidates when applying for leadership positions.

### Conflict of Interest

The authors declare that they have no conflicts of interest.
